# Executive Function and Falls in Older Adults: New Findings from a Five-Year Prospective Study Link Fall Risk to Cognition

**DOI:** 10.1371/journal.pone.0040297

**Published:** 2012-06-29

**Authors:** Anat Mirelman, Talia Herman, Marina Brozgol, Moran Dorfman, Elliot Sprecher, Avraham Schweiger, Nir Giladi, Jeffrey M. Hausdorff

**Affiliations:** 1 Movement Disorders Unit, Department of Neurology, Tel-Aviv Sourasky Medical Center, Tel-Aviv, Israel; 2 School of Health Related Professions, Ben Gurion University, Beer Sheba, Israel; 3 Department of Physical Therapy, Sackler Faculty of Medicine, Tel-Aviv University, Tel-Aviv, Israel; 4 Department of Neurology, Sackler Faculty of Medicine, Tel-Aviv University, Tel-Aviv, Israel; 5 Department of Behavioral Sciences, Academic College of Tel Aviv, Tel Aviv, Israel; 6 Harvard Medical School, Boston, Massachusetts, United States of America; Federal University of Rio de Janeiro, Brazil

## Abstract

**Background:**

Recent findings suggest that executive function (EF) plays a critical role in the regulation of gait in older adults, especially under complex and challenging conditions, and that EF deficits may, therefore, contribute to fall risk. The objective of this study was to evaluate if reduced EF is a risk factor for future falls over the course of 5 years of follow-up. Secondary objectives were to assess whether single and dual task walking abilities, an alternative window into EF, were associated with fall risk.

**Methodology/Main Results:**

We longitudinally followed 256 community-living older adults (age: 76.4±4.5 yrs; 61% women) who were dementia free and had good mobility upon entrance into the study. At baseline, a computerized cognitive battery generated an index of EF, attention, a closely related construct, and other cognitive domains. Gait was assessed during single and dual task conditions. Falls data were collected prospectively using monthly calendars. Negative binomial regression quantified risk ratios (RR). After adjusting for age, gender and the number of falls in the year prior to the study, only the EF index (RR: .85; CI: .74–.98, p = .021), the attention index (RR: .84; CI: .75–.94, p = .002) and dual tasking gait variability (RR: 1.11; CI: 1.01–1.23; p = .027) were associated with future fall risk. Other cognitive function measures were not related to falls. Survival analyses indicated that subjects with the lowest EF scores were more likely to fall sooner and more likely to experience multiple falls during the 66 months of follow-up (p<0.02).

**Conclusions/Significance:**

These findings demonstrate that among community-living older adults, the risk of future falls was predicted by performance on EF and attention tests conducted 5 years earlier. The present results link falls among older adults to cognition, indicating that screening EF will likely enhance fall risk assessment, and that treatment of EF may reduce fall risk.

## Introduction

The understanding of the relationship between age-associated declines in cognitive function and reduced mobility is evolving. For a long time, these two common geriatric symptoms were generally viewed as distinct and separate domains. Thus, fall risk in older adults – a major cause of morbidity and mortality [1]–[3] – was typically considered to be unrelated to age-associated changes in cognitive function. Whereas severe cognitive impairment in the form of dementia is known to increase the risk of falls [4]–[7], current guidelines advise that falls are only affected by cognitive function in this extreme case [1]. In the absence of dementia, there is, according to the established recommendations, no need to further assess the potential role of more subtle cognitive deficits. There is, however, reason to suspect that the relationship between cognitive function and falls is not one of “all or none” and that falls are affected by cognitive function even in the absence of dementia. There is a wide spectrum of age-associated changes in cognitive function that may also modify fall risk. Indeed, recent findings suggest that safe ambulation among older adults is more than a motor process, it also may involve executive function (EF) [8]–[11]. EF may be called in to play to compensate for age-associated decline in motor function and to allow for falls-free gait in complex, everyday situations that challenge an older adult's ability to walk while carrying out other motor and cognitive processes at the same time (e.g., talking to a companion during walking, reading a street sign, navigating an uneven surface, or planning ahead) and while inhibiting the response to potential distractions to gait (e.g., traffic noise). From this perspective, it seems only natural that EF deficits would exacerbate the risk of falls. Nonetheless, the degree to which EF contributes to future fall risk in dementia-free older adults has not yet been fully elucidated.

Retrospective and prospective reports suggest that falls and gait performance during dual tasking (DT), a commonplace, everyday activity, are related specifically to EF [12]–[14]. Indeed, DT walking may be considered a sub-domain or specific type of EF [8]–[11]. Falls status has also been associated with age-related changes in the prefrontal cortex and other brain regions that control EF [15], [16], further linking EF and falls. A number of reports have demonstrated that DT gait abilities predict future fall risk and that older adults with better EF, as quantified using cognitive testing, have a lower fall risk [12], [17], [18]. For example, we recently reported that EF predicted falls up to two years later [12]. In a study among cognitively intact community-living older adults, subjects with better EF at baseline were less likely to fall, compared to subjects who scored lower on the EF index. Our previous report was based on interim analyses, the overall number of falls was relatively small, and the follow-up period was confined to two years. Fall risk factors may, theoretically, change as the prediction interval becomes larger and age-associated changes in other factors are likely to diminish the value of testing of EF years earlier. It was important, therefore, to extend the period of follow–up and to evaluate how long the relationship between EF and falls exists.

In the present report, we investigated whether or not the relationship between EF and falls extends over a longer period in the same cohort. Evidence for an increased prodromal period would underscore the importance of cognitive function to fall risk. Substantiation of a long-term inter-dependence might also enhance our ability to detect fall risk well in advance of frequent falls, potentially allowing time for therapeutic interventions to take effect in a preventive manner. A relationship between EF and falls extended over several years would also provide more insight into the nature of this association. One could argue that over time, other factors will play more of a role and initial EF will no longer be associated with fall risk. On the other hand, we hypothesized that those subjects with better EF at baseline will be on a healthier trajectory and, therefore, will have a relatively low fall risk, even after 5 years. To address this question, we evaluated the ability of EF, as measured at baseline, to predict future fall risk over a follow-up period of 5 years. In secondary analyses, we examined the predictive value of other cognitive domains, putatively acting as “negative controls”, and of performance-based tests of gait and balance.

## Methods

### Participants

Recruitment of subjects from communities near Tel-Aviv, Israel was carried out as previously described [12], with testing spanning from February 2006-April 2007. Subjects were recruited from local senior centers via flyers, advertising, and word of mouth. In addition, a movement disorders specialist gave a serious of lectures on the potential link between gait disturbances, cognitive function and falls at local community centers. About 550 older adults expressed interest in participating in the study. An initial structured phone screen was used to identify community-dwelling, independent ambulators who were between the ages of 70 and 90 years and were free from disease likely to impact gait directly. This screening excluded subjects for a variety of reasons including age younger than 70 years old, use of a walking aid, or the presence of chronic disease. Subjects who passed the telephone screening were invited to the laboratory for a clinical examination and medical history. Subjects were included if they could walk independently and were free from disease likely to directly impact gait (e.g., vestibular, orthopedic) or fall risk (e.g., Alzheimer's, stroke, Parkinson's disease). Subjects were excluded if they had acute illness, history of brain surgery, major depression, or scored ≤25 on the Mini Mental State Examination (MMSE) [19]. The cohort included 256 community-living, healthy (at baseline) older adults [12]; thus a little less than 50% of the subjects who expressed interested were enrolled in the study. Baseline testing included thorough clinical and neurological evaluations and medical history. Subsequently, self-report and review of medical history were used to monitor major changes in medical status (e.g., the development of Parkinson's disease or Alzheimer's disease, as determined by the participant's clinician). The Charlson comorbidity Index was used for scoring general health status [20]. This index is a widely used metric that was developed to predict mortality for a patient who may have a range of co-morbid conditions such as dementia, cerebrovascular disease, heart disease, AIDS, and/or cancer (a total of 22 conditions). Each condition is assigned with a score of 1,2,3 or 6 depending on the risk of dying associated with this condition. The scores are summed up and given a total score which predicts mortality. 0 indicates no comorbidities.

### Ethics

The study was approved by the Helsinki committee at the Tel-Aviv Sourasky Medical Center. Informed written consent was obtained by all subjects prior to their entry into the study.

### Assessment of Falls

The number of falls in the year prior to the study, a predictor of fall risk [1], [21], was obtained based on self-report. Subsequently, prospective data on falls was collected using monthly calendars that were returned using pre-paid and pre-addressed envelopes, following current recommendations for the monitoring of falls [3]. Subjects were instructed to keep the calendar in a convenient place and to record falls, defined as unintentionally coming to rest on a lower surface [2], [3]. The circumstances and consequences of the fall were also recorded. Since the numbers of specific fall circumstances and consequences are relatively small, analysis of different fall types and consequences is beyond the scope of the present paper. On average, 60% of the falls diaries were returned on time by mail. Participants who failed to return the diary on time were contacted by telephone to obtain the missing data.

### Assessment of Cognitive Function

Cognitive function was evaluated at baseline using a previously validated, sensitive and reliable computerized neuropsychological assessment battery (MindStreams®, NeuroTrax Corp., TX) [22]–[26]. The EF index was based on computerized versions of the Go-No-Go and the Stroop interference tests, both related to response inhibition, and a “catch game” that assesses reaction time and errors in judgment on an eye-hand coordination task [27]. The assessment battery employed was designed to identify even subtle age-associated changes and covers a variety of cognitive domains, generating summary indices for EF, attention, memory and visual-spatial function and a global cognitive score (GCS). The EF and attention indices were derived from the same tests and are related constructs; the attention index may be viewed as a specific type of EF, largely a reflection of the ability to sustain attention. Each index and the GCS are summarized on an IQ-like scale, with 100 representing the estimated population mean normalized for age and education.

### Assessment of Gait, Balance and Dual Tasking Ability

Gait speed and gait variability were measured under two conditions: 1) “single task”, usual-walking at self-selected speed, and 2) DT, walking while subtracting 3 s from a predefined 3 digit number. Under each condition, subjects walked up and down a 25 meter-long, 2-meter wide hallway at their self-selected speed for two minutes while wearing force-sensitive insoles that enabled quantification of gait speed (mean over the middle 10 meters of the walk) and gait variability, specifically swing time variability, a property independent of gait speed that has been related to gait instability [12], [28]. The Berg Balance Scale [29], the Dynamic Gait Index [30], and the Timed Up and Go [31], [32] evaluated balance and functional mobility. A hand-held dynamometer measured grip strength (averaged over three attempts for the left and right hands) and lower extremity strength (quadricieps and tibilias anterior). The latter two measures were not associated with falls, hence, for brevity, we report only the results for grip strength, a widely used measure that reflects frailty and muscle strength in general. Depressive symptoms and level of fear of falling were evaluated using the Geriatric Depression Scale [33] and the Activities-specific Balance Confidence scale [34], respectively.

### Statistical Analysis

Statistical models were prepared using the total number of falls (as a count variable) per total follow-up time for each participant. To correct for over-dispersion, resulting in the underestimation of standard errors and overestimates of *X* ^2^ statistics, we used negative binomial regression (NBR) models with an offset variable for total months of follow-up [35], [36]. The NBR models estimated the influence of different predictors on the *rate* of falls, determining the rate ratio (RR) and their 95% confidence intervals (CI). Higher values, i.e., above 1.0, indicate a greater fall risk with increase in continuous predictors or different group-based predictors. When the RR is particularly small, larger increases (e.g., 10 units) rather than single unit increases are used to estimate the RR, e.g., rate(EF score +10)/rate(EF score) (no affect on level of significance). For RR based on group membership (e.g., gender), the RR is the ratio of fall rate associated with one group membership versus the other, e.g., rate(male)/rate(female). This method of interpretation also allows continuous and categorical predictors to be included in the same predictive model. The offset factor was included to correctly account for the number of months reported by each subject. NBR computes rate ratios over the entire period of observation for each individual predictor in the model, after adjusting for the influence of all other model predictors. For example, the inclusion of any falls that were reported in the year prior to baseline testing, age, and gender in the model will remove the influence of those factors from the effect of other model factors, such as EF, on future fall risk. Initially, each computerized battery cognitive index and the secondary outcome measures were entered separately into the analysis. Subsequently, significant independent factors were entered into multi-factorial models. Survival analyses using the Kaplan-Meier method assessed the impact of EF on time to first and second fall. Log-rank statistics evaluated the differences between groups defined by quartiles of the EF scores. Significance was accepted at p<.05. Analyses were performed using SAS and JMP (SAS Institute, Cary, NC).

## Results

Table 1 summarizes the baseline characteristics of the subjects. At study entry, mean scores were at or close to normative values for age on all tests of cognitive function, gait, mobility and affect. After the first two years of follow-up, seven subjects did not continue; they were diagnosed with stroke, Parkinson's disease (PD), Alzheimer's disease, major cognitive decline or lack of interest during this time period). In the third year of follow-up, eight subjects were dropped from the study (during this time period, two subjects passed away, one had a stroke, two were diagnosed with PD, one was diagnosed with Alzheimer's disease and two were not interested in continuing). Five subjects did not complete the fourth year of follow-up (one passed away, one stroke, two were not willing to continue). Thus, twenty subjects were lost to follow-up in the first 48 months after the baseline testing. During the follow-up period (maximum possible 66 months), the median number of months with falls data was 53 months (inter-quartile range 25%–75%: 49–58 months). A total of 570 falls were reported. 181 (71%) participants reported at least one fall during the follow-up period and among these, 118 (46%) participants reported more than one fall. 31% of subjects fell (range across the different follow-up years: 26–39%) in any given calendar year, with 11% falling more than 2 times per calendar year (range across the different follow-up years: 9–13%).

**Table 1:** Baseline characteristics of the subjects (n = 256).

Demographics, Affect & Grip Strength	Mean±SD (or %)
Age (yrs)* **^,##^**	76.4±4.5
Gender (female)**^##^**	61%
Years education (y)	13.67±3.48
Body-mass index (kg/m2)#	26.65±3.65
Charlson Comorbidity Index* **^,^** #(lower values reflect better health; 0 = none)	.8±1.1
Number of prescription medications	3.8±2.4
Reported no falls in the year prior to baseline testing**^##^** (%)	77%
Mini Mental State Examination (best possible score 30)	28.75±1.21
Activities Balance Confidence scale (%)* **^,##^**(max score 100; 0 = no confidence)	92.11±9.89
Geriatric Depression Scale* **^,##^**(max 30; 0 indicates no depressive symptoms)	5.25±4.71
Grip strength (kg)* **^,##^**	24.76±8.57

Computerized Cognitive Battery^x^	
Executive Function index	99.30±1.68
Attention index	99.03±12.74
Visual-Spatial index	97.05±15.86
Memory index	99.90±11.61
Global Cognitive Score	99.17±8.54

Gait and Mobility	
Berg Balance Scale(best possible score 56)	54.19±2.26
Timed Up and Go (sec)(above 13.5 seconds is considered abnormal)	9.48±1.61
Dynamic Gait Index(best possible score 24)	22.81±1.53
Usual-walking gait speed (m/s)	1.23±.22
Dual tasking gait speed (m/s)	1.10±.22
Usual-walking gait variability (%)	2.57±1.43
Dual tasking gait variability (%)	2.97±1.47

* Among the subject characteristics (i.e., demographics, affect, and grip strength), measures that were significantly correlated with EF are indicated, except for the Charlson index where the association was borderline (p = .068). The correlations were generally mild to moderate (i.e., |r| values less than .25; p<.01).

# and ^##^ indicate that this variable was marginally (.15>p>.05) or significantly associated with future fall risk, respectively, in univariate analysis among the measures listed under demographics. Univariate associations for the other measures are presented in Tables 2 and 3.

x Higher values indicate better performance on these computerized measures of cognitive function. 100 on these IQ-like scales represent the age and education adjusted norms.

In unadjusted models (Table 2), the EF and attention indices at baseline both independently predicted future fall risk (p<.001). When adjusting for age, gender, and fall history – well known predictors of falls [1], [21], [37], these indices continued to predict fall risk. When further adjusting for education (a gross measure of “premorbid” cognitive function, grip strength (a measure of muscle strength), and body-mass index (a measure reflecting frailty), the associations persisted (Table 2). Each additional 10 points on the EF index (i.e., indicating better performance) was associated with a 15% lower fall risk during the follow-up period. Similar results were observed for the attention index (Table 2). In contrast, the other cognitive tests were not significant predictors of future fall risk (p>.15). Scores on the Charlson Comoorbidity Index and the number of medications used were not significantly associated with future fall risk. Grip strength, body-mass-index and balance confidence (likely because this was related to history of falls) did not affect the association between the EF index and future fall risk.

**Table 2:** Cognitive measures and their ability to predict falls over the 66 months of follow-up.

	Unadjusted Model		Adjusted for age, gender, & fall history		Adjusted for age, gender, fall history, education, grip strength and BMI*	
	Rate Ratio (95% confidence interval)	P-value	Rate Ratio (95% confidence interval)	P-value	Rate Ratio (95% confidence interval)	P-value
EF Index	.79 (.69–.90)	.0005	.87 (.76–.99)	.037	.85 (.74–.98)	.021
Attention Index	.83 (.75–.93)	.001	.87 (.79–.97)	.013	.84 (.75–.94)	.002
Visual-Spatial Index	.93 (.85–1.02)	.152	1.00 (.92–1.09)	.989	1.02 (.92–1.12)	.743
Memory Index	.93 (.82–1.06)	.286	.97 (.86–1.09)	.617	.99 (.87–1.12)	.818
Mini Mental State Exam	1.05 (.32–3.47)	.938	1.88 (.60–5.87)	.277	1.38 (.41–4.60)	.596

* Rate ratios based on a 10 point change in each of the cognitive measures. Higher scores on the cognitive measures represent better performance and thus lower rate ratios represent a lower risk for falls. Because of (randomly) missing data for some tests, not all analyses included the same number of cases. Although it did not alter the conclusions, one subject, who met all study admission criteria and who was not otherwise atypical, was removed from NBR analysis because of his extremely high number of falls (49 falls) relative to all other subjects, and the ensuing disproportionate leverage his case had on the statistical models. His data were used for all other statistical tests. All of the results reported here and in Tables 3 and 4 were essentially unchanged if we also included the number of prescription medications in the fully adjusted model. BMI: body-mass index.

In unadjusted models, performance-based measures of mobility and DT gait speed all significantly predicted future fall risk (Table 3). When adjusting for age, gender and falls history, and in models that adjusted for other covariates, none of these measures significantly predicted future fall risk. In contrast, gait variability during DT still predicted future fall risk (p = .027).

**Table 3 pone-0040297-t003:** Performance-based measures of gait and mobility and their ability to predict falls over the 66 months of follow-up.

	Unadjusted Model		Adjusted for age, gender, & fall history		Adjusted for age, gender, fall history, education, grip strength and BMI*	
	Rate Ratio (95% confidence interval)	P-value	Rate Ratio (95% confidence interval)	P-value	Rate Ratio (95% confidence interval)	P-value
Berg Balance Scale	.92 (.86–.98)	.007	.96 (.90–1.02)	.163	.95 (.89–1.01)	.107
Dynamic Gait Index	.88 (.80–.97)	.011	.96 (.87–1.05)	.390	.97 (.87–1.07)	.499
Timed Up and Go	1.13 (1.03–1.24)	.007	1.07 (.97–1.16)	.161	1.08 (.98–1.18)	.115
Usual-walking Gait Speed	.57 (.27–1.19)	.136	1.20 (.56–2.58)	.643	1.55 (.66–3.6)	.310
Usual-walking Gait Variability	1.00 (.89–1.13)	.969	1.01 (.91–1.13)	.814	1.01 (.88–1.15)	.909
DT Gait speed	.40 (.20–.78)	.007	.67 (.33–1.34)	.256	.75 (.35–1.59)	.456
DT Gait Variability	1.14 (1.03–1.27)	.009	1.10 (1.00–1.21)	.054	1.11 (1.01–1.23)	.027

* In general, as expected, only age and history of falls were significantly associated with future fall risks. Note that in contrast to the results shown in Table 2, where higher values reflect better performance and lower risk of falls, for dual tasking gait variability and the Timed Up and Go, higher values indicate worse performance. Higher scores on these two measures were associated with an increased fall risk. During the 66 months of follow-up, 3 subjects were diagnosed with Parkinson's disease (1.1%), 4 with Alzheimer's disease (1.5%) and 2 sustained a stroke (.7%). The results summarized in Tables 2–4 were essentially unchanged when analyses were repeated after excluding these subjects.BMI: body-mass index.

In a combined EF and memory model (that included potential covariates), higher EF was significantly predictive of a lower fall risk (RR: .83, CI: .72–.97, p = .017), but memory was not (RR: 1.05, CI: .92–1.20, p = .507). Similarly, inclusion of MMSE scores had little impact on the association between EF and future fall risk (i.e., RR and p-values were essentially unchanged). In a model that evaluated EF and DT gait variability, EF was significantly associated with falls (RR: .85, CI: .73–.98, p = .024), while the association between DT gait variability and future fall risk became attenuated (RR: 1.09, CI: .99–1.20, p = .071), consistent with the idea that these two measures reflect similar underlying constructs. The individual parameters that contribute to the EF and attention indices were also examined. Slower reaction time, poorer accuracy and more errors on the Go-No-Go and catch game, each contributed to increased fall risk over the follow-up period (Table 4).

**Table 4:** Components that contribute to the EF and attention indices that were associated with falls over the 66 months of follow-up.*

	Mean +SD	Rate Ratio	95% Confidence Interval	P value
**Go-No-Go test**				
Accuracy (%)	90.92±11.48	.99	.97–1.00	.052
Response time (msec)	523.07±148.87	1.16	1.06–1.27	**.001**
(Accuracy/response time)*100	18.63±4.35	.94	.92–.98	**<.001**
Standard deviation of response time (msec)	151.43±120.44	1.19	1.07–1.34	**.001**
Commission errors	1.99±2.34	1.05	.99–1.12	.125
Omission errors	.89±2.11	1.07	1.00–1.14	**.049**
Response time for commission errors (msec)	463.42±351.44	1.06	1.01–1,11	**.012**
**Catch Game Test of hand-eye coordination**				
Direction changes	.38±.31	1.59	.99–2.55	.054
Accuracy**	480.06±219.56	.99	.99–1.00	.072
Errors	.74±.52	1.36	1.01–1.83	**.042**

* Each of these negative binomial regression models were adjusted for age, gender, years of education, BMI, history of falls and grip force. Only components that were significantly associated or tended to be associated (P<.15) with falls are shown. Rate ratios based on raw scores of these test components, except for the response time results which are reported based on 100 msec changes, instead of 1 msec; this transformation does not affect the p-value.

** Arbitrary units that reflect the total score (summed accuracy across sublevels, weighted by difficulty).

In univariate analysis, depressive symptoms, another potential fall risk factor, was a significant predictor of future falls (p<.01). The score on the Geriatric Depression Scale (GDS) was moderately correlated with EF performance (r = −0.23; p<.01). When tested together in a single model, EF and GDS both were significantly associated with future falls (p<.02), however, after adjusting for age, gender, and depressive symptoms, EF no longer predicted falls. Even after adjusting for GDS scores, attention and DT gait variability remained significantly associated with fall risk.

**Figure 1:**
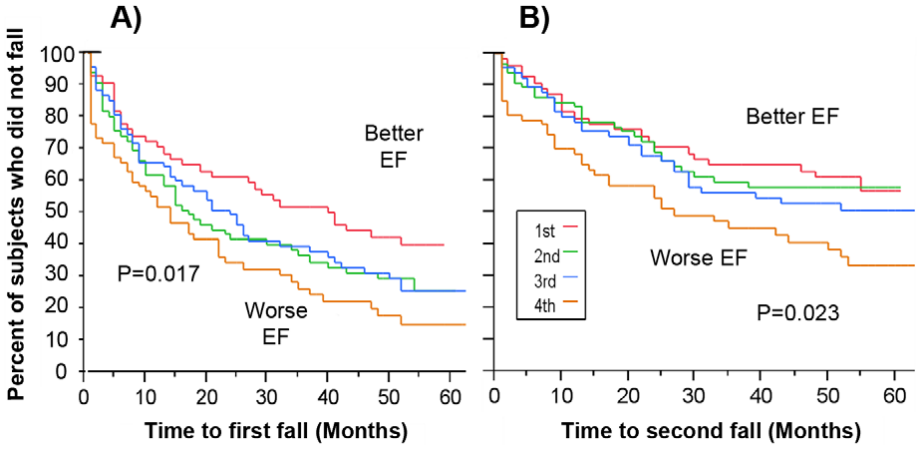
Survival curves illustrating the percent of subjects who did not fall as a function of time and executive function (EF). Differences were found in ‘time to first fall’ between the highest (indicated as 1^st^ on the graph) and lowest EF (indicated as 4^th^ on the graph) quartile (P = 0.017) and time to second fall (P = 0.023). Subjects with in lowest quartile were more likely to fall sooner (LEFT) and more likely to become multi-fallers sooner (RIGHT) than those in highest quartile. Note when performing similar analyses on those subjects who reported no falls in the year prior to the study, subjects with lowest quartile of EF were also more likely to fall during the follow-up period, similar to what is observed if the entire cohort is included in the analysis. EF quartile was defined based on the ranking of EF scores obtained at baseline using the computerized cognitive battery. By definition, subjects in the lowest quartile had the lowest (i.e., relatively worst) EF scores, whereas subjects in the highest quartile had the highest (i.e., best) scores.

Survival analysis showed that when participants were stratified into EF quartiles, subjects in the lower quartile were more likely to fall during the course of follow-up (p = .002), compared to those subjects in the highest EF quartile (see Figure 1) and were more likely to become multiple fallers sooner (p = .023) (see Figure 1b). The middle two groups correspond to 50% of the subjects whose scores most closely represent the mean of the population. Differences were found in time to first fall between the lowest quartile and the middle two quartiles (p = .044) and there was a trend when comparing survival of the top EF quartile and the middle two quartiles (p = .072). Of note, among all fallers, 51% of those in the lower EF quartile were multiple fallers compared to only 36% in the other three quartiles (p = .043).

## Discussion

The primary aim of this study was to determine the relationship between EF and falls in older adults during five years of follow-up. The findings support our hypothesis and demonstrate that among community-living older adults, the risk for future falls was predicted by EF and attention tests conducted five years earlier. The negative binomial regression analyses adjusted for previous falls; thus, this timeline supports the possibility that specific cognitive deficits lead to an increased risk of falls, and that the association between EF and future falls is not simply a by-product of parallel processes. By taking into account any falls in the year prior to the baseline testing, we can infer that a relatively poor EF score preceded an increased fall risk. Nonetheless, cause and effect cannot be concluded from this observational study.

The relationship between EF and future falls could be explained in several ways. EF could simply be a general marker of cognitive aging, but the results do not support this interpretation. If broad age-related cognitive function were driving the results, one would have anticipated that the MMSE and memory index would also be related to future fall risk, however, this was not the case. Memory and the MMSE did not predict future fall risk and these cognitive measures did not modify the association between EF and future fall risk. While some studies suggest that memory may play a role in fall risk [38], the present findings are consistent with previous studies that showed that MMSE and memory are generally not strongly associated with gait and fall risk among non-demented older adults [11], [13], [18], [39], [40]. Previous studies have suggested that depression may contribute to fall risk [41], [42], leading to the idea that there is a complex interplay between depression and falls [42]. The present findings support this idea (e.g., EF and depressive symptoms were associated with each other and they both predicted future falls). Nonetheless, the results also suggest that the association between cognitive function (e.g., dual tasking gait variability and attention) and future falls persists even after taking account depressive symptoms. Thus, depressive symptoms do not fully explain the relationship between cognitive function and falls observed herein.

Another possibility is that older adults with better EF more capably deal with challenging walking conditions that require higher-level cognitive control and DT abilities [12], [13], [23]. The results are consistent with this possibility. Indeed, motor tests that reflect usual walking and balance abilities (e.g., gait speed, Berg Balance Scale) were not predictive of falls once age, gender and a history of falls were taken into account. Among the performance based measures of gait, balance and mobility, only DT gait variability predicted future fall risk (in models that adjusted for age; recall Table 3). The survival analyses findings (Figure 1) also suggest that better EF may ‘shield’ older adults from falling, enabling a subject to appropriately allocate the necessary cognitive resources to maintain balance during walking and prevent or recover from any disturbances. Alternatively, perhaps, relatively poor EF restricts the ability to compensate for age-associated changes in gait and balance, hence increasing the risk of falls.

At baseline, all subjects had relatively intact cognitive function (e.g., MMSE>25). Nonetheless, there was a range in EF abilities. This result is consistent with recent reports of reduced frontal lobe connectivity to functionally linked cortical areas [43]–[46] and lower accuracy on EF-related tasks, even among older adults who show no overt signs of cognitive decline [47]. These findings converge with the frontal aging hypothesis [48] and point to the possibility that frontal lobe dysfunction can be thought of as a decrease in cognitive reserve due to specific changes in the brain. These reports [43]–[46] and the present results support the possibility that as this cognitive reserve declines with aging, the impact of EF on falls becomes more prominent.

In our earlier study in the same cohort, EF was related to falls after two years [12]. Further, grip strength, a measure of frailty, and DT gait variability were associated with falls in multivariate analyses. Over the extended 66 months of observation, DT gait variability and EF continued to be associated with fall risk, but grip strength was no longer significantly related to future fall risk (p>.53, data not shown). This supports the idea, as mentioned in the Introduction, that predictors of falls may change as the observation period is increased. The observed associations are consistent with the idea that both reflect similar, probably frontal functions, tested here in two different ways. In other words, DT abilities represent, in part, motor-cognitive dependence and EF competence, so that they both predict falling. Indeed, several studies have demonstrated that EF contributes to the impact of DT on gait [11], [17], [18].

Closer examination of the components of EF and attention reveals that poorer accuracy, more errors, and longer response time on tests of response inhibition were related to future fall risk (Table 4). These EF constructs are critical for everyday walking and DT gait [18]. When actively engaged in mobility tasks during daily life, distractions that compete for attention and require inhibition often appear. The delayed reaction time on EF tasks, a deficit that may limit the ability to quickly respond to loss of balance [49], and the association between gait during DT and falls in the present study underscores the importance of attention and EF in the safe mobility of older adults. In part, falling apparently results from a decline in the ability to efficiently negotiate with the environmental stimuli and potential obstacles at the same time. As walking requires more mental effort with advancing years, the decline in EF may make an individual more prone to distractions while walking and perhaps less competent in the motor-cognitive coordination involved, thus increasing fall risk.

This study has several limitations. For example, the participants in the present cohort may not represent aging in general, but may reflect what occurs in more successful aging. Nevertheless, the percent of subjects in this study who reported a fall each year was very similar to the 33% fall rate widely reported in the literature [1], [4], [28] and the mean values reported in Table 1 are all consistent with those of healthy older adults (e.g., the computerized cognitive test mean values are essentially identical to 100, the value anticipated for age-matched norms). Further the absence of major motor and cognitive co-morbidities may have enabled the unmasking of the role of EF in the predisposition to falls. We also did not measure all of the factors that have been associated with falls in the past. For example, previous studies have demonstrated that pain is a predictor of falls [36], [41] and lack of information concerning pain and other potential mediators of the observed relationships could be considered a limitation of the current study. Strengths of the present investigation include the use of a standardized computerized battery covering several cognitive domains, the quantitative assessment of gait during single and DT, and consistent findings using two different statistical approaches. Another strength of the study is the fact that it prospectively examined the association between baseline cognitive function and falls based on monthly calendars, the recommended method, over a relatively long time period, with more than 75% of the subjects reporting more than 49 months of fall reports.

Current clinical practice does not call for the assessment of EF, attention or dual tasking abilities when evaluating fall risk. Most studies designed to assess fall risk either do not test cognitive function at all or use only general screening measures like the MMSE, which may not be sufficiently sensitive [37]. Even the recently updated guidelines for the prevention for falls in older persons found that there is insufficient evidence for supporting any cognitive recommendations for assessment or reduction of fall risk [1], while making no distinction between cognitive domain sub-types or EF. The present findings are consistent with earlier work [12], [13] which suggests that EF is related to falls. Here we extend those findings by demonstrating that the assessment of EF and attention can provide the clinician with important information about the risk of falling that may remain undetectable during a routine motor or cognitive screening, potentially providing several years of advanced warning. Perhaps, it is time to give more credence to the possibility that tests of EF and attention can augment the early identification of subjects who are likely to have an increased fall risk in the future and to incorporate testing of these cognitive domains into screening batteries. The results of the present study are also consistent with the intriguing possibility of a cause and effect relationship between EF deficits and fall risk. If that is indeed the case, then interventions designed to improve EF and DT abilities will decrease the risk of falls as suggested by a handful of pilot studies [50]–[52]. Additional work is, however, needed to more fully evaluate this possibility.
